# Cesarean Scar Pregnancy Management: Minimally Invasive Suction of the Gestational Sac Content Combined With Local and Intramuscular Methotrexate Injection

**DOI:** 10.31486/toj.20.0121

**Published:** 2020

**Authors:** Feras Sendy, Samer J. Ahmeed, Sulaiman Al Obaid, Bahaaldden Sallout, Sameer Sendy

**Affiliations:** ^1^ Department of Obstetrics and Gynecology, CHU Estaing, Clermont-Ferrand, France; ^2^ Faculty of Medicine, Université Clermont Auvergne, Clermont-Ferrand, France; ^3^ Department of Obstetrics and Gynecology, King Fahad Medical City, Riyadh, Saudi Arabia; ^4^ Department of Obstetrics and Gynecology, Dr. Abdul Rahman Al Mishari Hospital, Riyadh, Saudi Arabia

**Keywords:** *Hemorrhage*, *methotrexate*, *pregnancy–ectopic*, *suction*, *ultrasonography–prenatal*

## Abstract

**Background:** Cesarean scar pregnancy is a rare, potentially life-threatening complication in patients with prior cesarean delivery. Vaginal bleeding is a common presenting symptom.

**Case Report:** A 23-year-old female who presented with mild vaginal bleeding was diagnosed by transvaginal ultrasound with a viable cesarean scar pregnancy of 7 weeks’ gestation. After the sac content was suctioned through a transvaginal approach under ultrasound guidance, the patient was injected with 50 mg local and 25 mg systemic methotrexate. One week later, a repeat systemic methotrexate dose of 50 mg was administered. The patient's beta human chorionic gonadotropin (hCG) levels were followed weekly until a negative beta hCG level was established.

**Conclusion:** No management approach has been universally approved for cesarean scar pregnancy; the best option depends on case presentation, surgeon experience, and available facilities. We suggest that our minimally invasive treatment is an acceptable approach, especially if embryonic cardiac activity is present. We recommend the referral of such cases to tertiary centers to avoid complications.

## INTRODUCTION

Cesarean scar pregnancy is a rare but dangerous potential complication for patients with a history of cesarean delivery. In 2017, the estimated rate of cesarean delivery in the United States was 32%.^1^ The estimated prevalence of cesarean scar pregnancy is 1/1,800 to 1/2,500 of all cesarean deliveries performed, but the true incidence of cesarean scar pregnancy is unknown.^2-4^ The majority of cases are associated with a history of cesarean delivery. Vaginal bleeding is a common presenting symptom, yet asymptomatic patients are diagnosed incidentally via ultrasonography. Early diagnosis in the first trimester is crucial so patients can be counseled about the risks of cesarean scar pregnancy and appropriate management can be implemented. With early detection, many patients, depending on their clinical stability, can be treated in an office setting without general anesthesia.

We report a case of a viable cesarean scar pregnancy managed by transvaginal suction of the sac content and simultaneous injection of local and systemic methotrexate under ultrasound guidance.

## CASE REPORT

**Figure 1.**
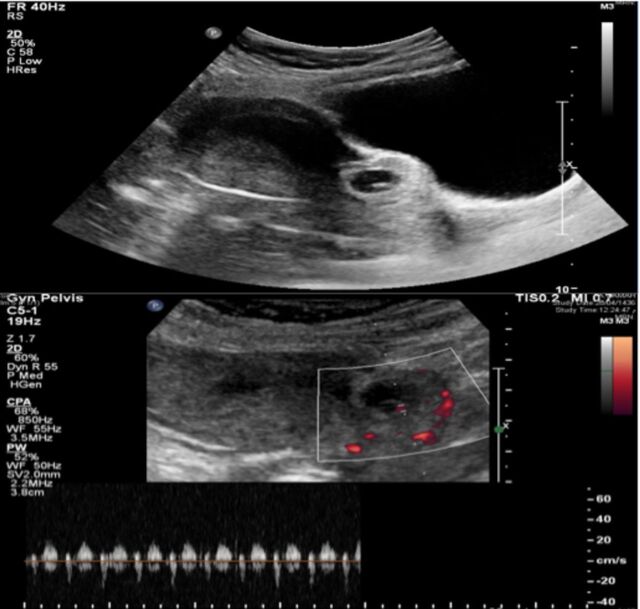
**Transvaginal ultrasound shows gestational sac with a viable embryo on the lower part of the uterus at the site of the cesarean section scar.**

A 23-year-old female gravida 2 para 1 was referred to our hospital with a 1-week history of mild vaginal bleeding. She had already been diagnosed with a cesarean scar pregnancy via transvaginal ultrasonography and magnetic resonance imaging (MRI) that showed the pregnancy 2.5 mm from the bladder wall. Two years prior to presentation, the patient had had a cesarean delivery for macrosomia without complications. Repeat ultrasound at the current presentation showed a single viable gestational sac corresponding to 7 weeks’ gestation at the cesarean scar site (Figure 1). Endometrial thickness was 12 mm, and blood was seen in the uterine cavity at the level of the fundus. Abdominal examination was unremarkable. Speculum examination revealed minimal blood in the vagina. Laboratory workup showed normal hemoglobin level, platelet count, white blood count, and C-reactive protein. After the risks of cesarean scar pregnancy and benefits of treatment were explained, the couple chose termination of pregnancy; informed consent was obtained.

The patient was positioned similarly to infertility patients for office-based ovum pickup. A transvaginal probe fitted with a suction double lumen needle (Kitazato Corp) was introduced into the vagina, and the sac content was aspirated until no cardiac activity was detected. Methotrexate 50 mg was injected into the sac, and a 25-mg methotrexate injection was administered intramuscularly. The patient was hospitalized for 1 week after the procedure. During the first 2 days postoperatively, the patient had minimal vaginal spotting associated with mild lower abdominal pain. Her vital signs were stable throughout her hospital stay, and she was discharged on postoperative day 7.

**Figure 2.**
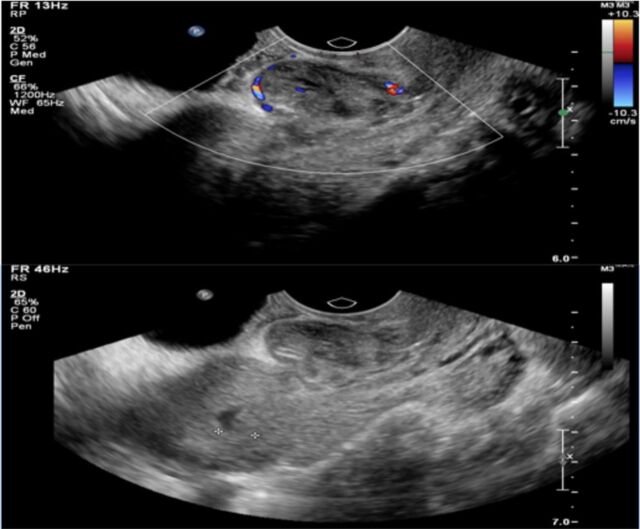
**Transvaginal ultrasound after minimally invasive medical treatment shows a well-defined mass that is inhomogeneous in texture with surrounding vascularity and a small collapsed gestational sac without a yolk sac or embryo at the site of the scar.**

One week after the procedure, transvaginal ultrasound showed a well-defined soft tissue mass that was inhomogeneous in texture with surrounding vascularity at the site of the cesarean scar and a small collapsed gestational sac without a yolk sac or embryo (Figure 2). Beta human chorionic gonadotropin (hCG) was measured on postoperative days 1, 4, and 7 and increased on day 4, followed by a decline of 16.4% on day 7. Another 50 mg of methotrexate was administered intramuscularly 1 week after the procedure, and the patient was followed on an outpatient basis for 2 weeks with weekly beta hCG surveillance until a negative beta hCG level was established. The patient reported no side effects or complications.

## DISCUSSION

Cesarean scar pregnancy most commonly occurs when a pregnancy implants on a cesarean delivery scar. The exact mechanism is not well understood, but one hypothesis is that the blastocyst implants over fibrous scar tissue within the myometrium of the anterior wall of the lower uterine segment.^4^ Cesarean scar pregnancy is also, but less frequently, associated with uterine surgery, such as curettage, myomectomy, endometrial ablation, manual removal of the placenta, or other intrauterine surgical manipulation.^4^ Our patient had a history of cesarean delivery. Esposito et al examined the interpregnancy interval in relation to uterine scar failure in a case-control study and determined that an interpregnancy interval <6 months was highly associated with uterine scar failure.^5^ One possible inference from this finding is that cesarean scar pregnancy may be indirectly related to interpregnancy interval.

Cesarean scar pregnancy has 2 types. In the first type, the sac implants on a scar, and the pregnancy progresses to the cervico-isthmus space and the uterine cavity.^4^ This type of cesarean scar pregnancy can progress until viability, but rupture is a risk. In the second type of cesarean scar pregnancy, deep implantation occurs in a cesarean scar defect, called a niche, with progression toward and risk of rupture and adherent placenta higher than with the first type.^4^ Myometrial thickness between the placenta/gestational sac and the anterior uterine surface of the bladder is measurable in the first type of cesarean scar pregnancy.^6^ In the second type, the placenta/gestational sac complex is proximate to the bladder or the anterior uterine surface.^6^ Kaelin Agten et al showed the importance of myometrial thickness in predicting outcomes. Myometrial thickness of ≥4 mm was associated with higher gestational age and neonatal birth weight at delivery and lower risk of bleeding, cesarean hysterectomy, and morbidly adherent placenta at delivery.^6^ If the myometrial thickness was ≤2 mm, the risk of an adherent placenta leading to cesarean hysterectomy was increased.^6^ Measuring myometrial thickness during the first trimester could possibly help to diagnose patients with an adherent placenta.

While cesarean scar pregnancy can be diagnosed by transvaginal ultrasonography or MRI, transvaginal ultrasound appears to be the best first-line imaging modality for diagnosis.^4^ Timor-Tritsch et al asserted that transvaginal ultrasonography during the first trimester provides an accurate diagnosis without the need for other imaging modalities, using the following 4 diagnostic criteria: an empty uterine cavity and a closed and empty endocervical canal; an early gestational sac and/or placenta proximate to the hysterotomy scar/niche with fetal or embryonic pole and/or yolk sac with or without a heartbeat; absent or thin-appearing myometrial layer between the gestational sac and the anterior uterine wall or the bladder wall; and abundant blood flows around the gestational sac determined by Doppler interrogation.^4^ In our case, transvaginal ultrasonography was sufficient to obtain adequate diagnostic information, although our patient underwent MRI in another hospital before referral, possibly to confirm the diagnosis.

According to a 2012 review, 13.6% of cesarean scar pregnancy diagnoses were missed, leading to hysterectomy and consequent loss of fertility in some cases.^7^ Complications of cesarean scar pregnancy include development of adherent placenta, uterine rupture, severe hemorrhage, prematurity, and cesarean hysterectomy.^8,9^

In their review of the literature from 1972 to 2011, Timor-Tritsch and Monteagudo identified 31 treatment modalities used in 645 cases of cesarean scar pregnancy.^7^ They found that gynecologists tend to use surgical approaches such as dilation and curettage (D&C) and laparoscopy or hysteroscopy, whereas obstetricians tend to prefer medical treatment such as methotrexate injection and uterine artery embolization.^7^ Uterine artery embolization or D&C alone had a range of complications up to 80%. D&C and uterine artery embolization as a primary modality combined with other treatments resulted in a complication range up to 100%. Intramuscular methotrexate injection combined with D&C had a complication rate of 86%. On the other hand, hysteroscopy as a primary treatment approach had a complication rate of 18.4%, while local methotrexate injection had the lowest complication rate of 9.6%.^7^ The authors found from their review of 184 pregnancies of 5 to 15 weeks’ gestation that early diagnosis and treatment vs delayed diagnosis and treatment tended to result in better outcomes, defined as the lack of complications (heavy bleeding, embolization, and emergency surgery).^7^ Thus, gestational age at diagnosis is directly proportional to the risk of complications. A possible reason for the high complication rates associated with D&C is the limited myometrial layer/scarred tissue in the lower uterine segment. Uterine artery embolization is a symptomatic rather than a curative treatment as the vascular obstruction is temporary. Hysteroscopy appears to be a promising modality for experienced gynecologists. However, generalizing such a recommendation based on a limited number of cases is problematic. Local injection of methotrexate seems to be associated with good outcomes such as lower risk of heavy bleeding, embolization, and emergency surgery, possibly because of its direct action on the gestational sac and providers’ familiarity with its use for pregnancy reduction and ectopic pregnancies. In our case, we used the double lumen needle (Kitazato Corp) to mechanically disrupt the pregnancy followed by combined methotrexate injections, local and intramuscular, to maximize the effectiveness.

The objective of a 2016 retrospective case series was to evaluate the effectiveness of the double cervical ripening balloon in preventing bleeding in the termination of 7 cesarean scar pregnancies and 3 cervical pregnancies.^10^ The hypothesis was that inflating the upper balloon into the uterine cavity would help prevent expulsion, while the lower balloon positioned opposite the gestational sac would stop the blood supply of the gestational sac and prevent bleeding. The treatment was effective, as only 1 patient experienced bleeding. The median time from treatment to total beta hCG drop was 49 days. Limitations of the study were the small number of patients and that the balloon was left in place for up to 5 days.^10^

The case series was expanded to treat 38 patients with double cervical ripening balloon combined with systemic methotrexate treatment.^11^ One patient had a hysterectomy because of excessive bleeding 27 days after the procedure, but treatment for all the other patients was successful and without complications. In the expanded series, the time of balloon placement was reduced to 1 day.

Given the rarity of cesarean scar pregnancy and the difficulty of comparing studies, use of a double cervical ripening balloon combined with systemic methotrexate may be the future of treating cesarean scar pregnancy, as the balloon appears to prevent bleeding in many cases. Theoretically, our technique could have posed a higher risk of bleeding given the hypothesis of the balloon placement, but our unique modality of suction via double lumen needle under transvaginal ultrasound guidance combined with local and systemic methotrexate injection was successful.

## CONCLUSION

No management approach has been universally approved for cesarean scar pregnancy. Determining the best option depends on case presentation, surgeon experience, and available facilities. We recommend referral of such cases to tertiary centers for management to avoid complications. Multicenter evaluation of treatments for cesarean scar pregnancy is necessary.

## Notes

This article meets the Accreditation Council for Graduate Medical Education and the American Board of Medical Specialties Maintenance of Certification competencies for Patient Care and Medical Knowledge.
